# DHX37 protein and mRNA expression patterns in breast and ovarian cancer and their prognostic implications

**DOI:** 10.1007/s00418-026-02491-9

**Published:** 2026-05-18

**Authors:** Denys Ndeke, Sandra Sam, Nuanphan Polchai, Khalid Alshammari, Megan R. Greener, Tangkam R. Marak, Jimmy Joyce, Maisie Henman, Suha Deen, Andrew R. Green, Ian O. Ellis, Emad A. Rakha, Stewart G. Martin, Sarah J. Storr

**Affiliations:** Nottingham Breast Cancer Research Centre, School of Medicine, University of Nottingham Biodiscovery Institute, University Park, Nottingham, NG7 2RD UK

**Keywords:** Breast cancer, Ovarian cancer, DHX37, Prognosis

## Abstract

DEAH-box RNA helicase 37 (DHX37) is a highly conserved RNA helicase involved in ribosome biogenesis, RNA metabolism and immune regulation. Dysregulation of RNA helicases has been implicated in tumourigenesis; however, the clinical significance of DHX37 expression in tumours is still emerging and requires further study. This study investigated the expression of DHX37 in large breast and ovarian cancer patient cohorts using immunohistochemistry in 1512 breast and 420 ovarian cancer cases and complementary transcriptomic datasets. In breast cancer, low cytoplasmic DHX37 protein expression was significantly associated with adverse clinicopathological parameters including larger tumour size, higher grade, lymphovascular invasion and positive nodal status (all *P* < 0.001). Low DHX37 expression was associated with poor breast cancer-specific survival (*P* < 0.001), particularly among oestrogen receptor-positive patients. In ovarian cancer, low DHX37 expression was associated with lower FIGO stage, absence of residual disease and improved overall survival (*P* = 0.013). DHX37 shows contrasting prognostic associations in breast and ovarian cancer, suggesting context-dependent biological functions. Reduced DHX37 expression may promote tumour progression in ER-positive breast cancer, but is associated with favourable outcomes in ovarian cancer. These findings highlight DHX37 as a potential prognostic biomarker and underscore the need for functional studies to elucidate its mechanistic role in tumour biology and immune modulation.

## Introduction

DHX37 is a highly conserved DEAH box RNA helicase that uses ATP-driven 3′-5′ translocation to remodel ribonucleoprotein complexes. It plays a vital role in ribosome biogenesis by facilitating the processing of 18S ribosomal RNA (rRNA) in the early pre-rRNA processing stage and assembly of the small ribosomal unit. The activity of DHX37 is tightly regulated by co-factors such as UTP14A, which contains a conserved sequence motif that directly activates DHX37 by stimulating its ATPase activity to enhance RNA binding. Depletion of DHX37 leads to the accumulation of pre-ribosomal intermediates, ultimately compromising translational capacity (Boneberg et al. 2019; Choudhury et al. 2019).

Beyond ribosome biogenesis, DHX37 and other DEAH box proteins participate in RNA metabolism, including RNA splicing, surveillance, transport and decay (Jankowsky 2011). Dysregulation of RNA helicases, including DHX37, has been linked to disruptions in cellular homeostasis and increased susceptibility to various diseases, including cancer (Devasahayam Arokia Balaya et al. 2025). In the immune system, DHX37 has been implicated as a negative regulator of CD8^+^ T cell activity. Genome-wide Clustered Regularly Interspaced Short Palindromic Repeats (CRISPR) screens have shown that DHX37 interacts with NF-κB pathway components to suppress T-cell activation and effector functions (Dong et al. 2019). Collectively, these findings highlight DHX37 as a multifunctional RNA helicase which plays pivotal roles in ribosomal biogenesis, metabolism and immune modulation emphasising its importance in maintaining cellular homeostasis.

Pathogenic variants of DHX37 have been associated with human ribosomopathies, a class of clinical conditions that are caused by defects in ribosome biogenesis. In particular, DHX37 has been implicated in 46, XY disorders of sexual development (DSD). Exome sequencing studies from individuals with 46, XY DSD have identified recurrent missense mutations in the *DHX37* gene (*n* = 52). Strong genetic associations have been observed in these studies between pathogenic variants in conserved amino acid residues within functional regions of DHX37 and two types of DSD: non-syndromic 46, XY DSD and 46, XY testicular regression syndrome (Buonocore et al. 2019; da Silva et al. 2019; McElreavey et al. 2020; Zidoune et al. 2021). These findings suggest that DHX37-associated DSD constitutes a novel form of ribosomopathy. The precise mechanisms underlying DHX37-associated DSDs remain poorly understood, although ribosome synthesis and the Wnt and NF-κB pathways may be affected (Peng et al. 2025). Furthermore, a *DHX37* gene mutation has been implicated as a cause of azoospermia in a male patient with Sertoli cell-only syndrome (Al-Hadyan et al. 2024).

Variants of several DEAH-box RNA helicases are associated with congenital neurodevelopmental disorders (Yamada et al. 2023). Variants in DHX37 have been implicated with global developmental delay and intellectual disability, central nervous system anomalies and vertebral defects (*n* = 13). Some of these phenotypes are also seen in variants of DEAH-box RNA helicases indicating convergence of disease processes (Fiorenzani et al. 2025; Paine et al. 2019). Given its broad roles in RNA metabolism and immune regulation, aberrant DHX37 activity could contribute to tumourigenesis by enhancing translational capacity, deregulating oncogenic transcription and modulating tumour-immune interactions.

*DHX37* has recently emerged as a gene of interest in oncogenesis owing to its role in RNA processing and assembly; processes which are frequently dysregulated in cancer. Bioinformatic analyses have shown that *DHX37* mRNA is upregulated in several tumour types, including breast, colon, hepatocellular, lung, prostate and gastric cancers (Huang et al. 2021). High *DHX37* expression was also linked to upregulation of cancer-related pathways supporting the notion that DHX37 may drive oncogenesis in various cancers (Huang et al. 2021).

High DHX37 expression has been consistently associated with worse overall survival, disease-free survival and progression-free survival time in hepatocellular carcinoma (HCC) and lung adenocarcinoma (Huang et al. 2021). DHX37 may contribute to tumour progression of HCC and lung adenocarcinoma through immune cell infiltration and fostering an immunosuppressive environment thereby facilitating tumour progression (Xu et al. 2020). Furthermore, DHX37 can collaborate with PLRG1 to activate CCND1 (cyclin D1) transcription via promotor and super-enhancer elements, driving cell proliferation and contributing to the poor prognosis of patients with HCC (Liu et al. 2022). These findings highlight DHX37 as a critical factor that promotes tumour progression through transcriptional activation and immune modulation, emphasising its potential as a prognostic marker and therapeutic target.

Emerging research also implicates DHX37 in breast and ovarian cancers. In breast cancer, transcriptomic analyses have demonstrated significant association between upregulation of DHX37 and invasive features and reduced relapse-free and overall survival (Huang et al. 2021). Genome-scale in vivo CRISPR screens in mouse models of triple negative breast cancer identified DHX37 as a negative regulator of CD8^+^ T cell activity, where its deletion enhanced tumour infiltration and cytotoxicity, demonstrating a potential immune-modulatory role (Dong et al. 2019). In ovarian cancer, DHX37 is significantly upregulated compared with normal tissue but its prognostic significance is unclear. Despite emerging evidence linking DHX37 to cancer progression, its protein-level expression and prognostic value have not been systematically evaluated in large, clinically annotated patient cohorts. Moreover, the potential context-dependent roles of DHX37 across hormonally driven and non-hormonally driven tumours remain unexplored. This study therefore aimed to determine DHX37 protein expression in large, well-defined cohorts of breast and ovarian cancer and to investigate its associations with clinicopathological features, molecular subtypes and patient outcomes.

## Methods

### Breast cancer patient cohort

Patients who received treatment at Nottingham University Hospitals between 1998 and 2006 with early-stage invasive breast cancer were available for assessment (*n* = 1512). All patients underwent either mastectomy or breast conserved surgery depending on disease characteristics or patient choice with adjuvant radiotherapy if indicated. Adjuvant systemic treatments were based on Nottingham Prognostic Index (NPI), oestrogen receptor status and menopausal status. Patients with NPI scores of 3.4 or above were indicated for adjuvant chemotherapy with cyclophosphamide, methotrexate and 5-fluorouracil regimens if they were also ER-negative subtype or pre-menopausal; whereas patients with an NPI score below 3.4 did not. ER-positive patients were administered hormonal therapy. No patients were given trastuzumab. Studies using this patient cohort have been published previously (Lacey et al. 2024; Saidy et al. 2025, 2023).

### Ovarian cancer patient cohort

Patients who received treatment at Nottingham University Hospitals between 1991 and 2011 with ovarian cancer were available for assessment (*n* = 420). Patients were treated with adjuvant chemotherapy, with over 60% of patients receiving platinum-based chemotherapy. Clinicopathological data of patients were collected and included the age, stage of the cancer, histological subtype, grade, type of treatment and survival outcomes. Studies using this patient cohort have been published previously (Kobel et al. 2023; Vasan et al. 2023).

### TCGA patient cohorts

Gene expression data were obtained from the TCGA PanCancer Atlas breast cancer and ovarian cancer cohort and downloaded from cBioPortal and NCI Genomic Data Commons (GDC) (Cancer Genome Atlas Research et al. 2013; Cerami et al. 2012; Gao et al. 2013; Heath et al. 2021). Data analysis was performed on mRNA expression *z*-scores relative to diploid samples (log microarray). To assess tumour mRNA expression vs matched adjacent normal tissue, samples were annotated using TCGA barcodes and classified as primary tumour or solid tissue normal with patients with matched tumour—normal pairs retained for this analysis; this assessment was only performed in the TCGA breast cancer cohort owing to the very low numbers of matched pairs available in the TCGA ovarian cancer cohort.

### Western blotting

Cell lysates, from T47D and MDA-MB-231 breast cancer cell lines, were prepared using radioimmunoprecipitation assay (RIPA) buffer supplemented with ethylenediaminetetraacetic acid (EDTA) and protease/phosphatase inhibitor cocktails (Thermo Scientific, Cheshire, UK). Samples were mixed with Bolt LDS sample buffer and reducing agent (Invitrogen, Paisley, UK) and denatured at 100 °C for 10 min. Protein separation was carried out on Bolt 4—12% Bis–Tris Plus gels (Invitrogen, Paisley, UK) using the Mini Gel Tank system and Bolt MES SDS running buffer at 200 V for 30 min. Proteins were transferred onto 0.45 µm nitrocellulose membranes (Amersham Protran, Cytiva, Buckinghamshire, UK) in Bolt transfer buffer (Invitrogen, Paisley, UK) containing 10% methanol at 10 V for 1 h. Membranes were then blocked for 1 h at room temperature in 5% non-fat milk prepared in phosphate-buffered saline (PBS) containing Tween-20 (Sigma-Aldrich, Dorset, UK). Blots were incubated overnight at 4 °C with primary antibody against DHX37 (AB70778 1:1000, Abcam, Cambridgeshire, UK), followed by incubation with donkey-anti-rabbit secondary antibody (926-32213; 1:5000, Li-Cor, Cambridgeshire, UK) for 1 h at room temperature. Signal detection was performed using the Odyssey FC Imager (Li-Cor, Cambridgeshire, UK), and images were analysed with Image Studio software (version 4.1).

### Immunohistochemistry

Breast and ovarian tissue microarray Sects. (4 μm thick) were deparaffinised in xylene and rehydrated through ethanol to water. Antigen retrieval was performed by heating the sections in 0.01 M sodium citrate buffer (pH 6.0) in a microwave for 20 min. Immunostaining was carried out using the Novolink Polymer Detection System (RE7150-K, Leica Biosystems, Buckinghamshire, UK) following the manufacturer’s protocol. DHX37 antibody (AB70778, 1:100, Abcam, Cambridgeshire, UK) was incubated on the sections for 1 h at room temperature. After antibody incubation, sections were washed with Tris-buffered saline (TBS), prior to incubation with Novolink Post Primary solution, TBS and then Novolink Polymer Solution. Sections were then dehydrated using ethanol, cleared in xylene and mounted with dibutylphthalate polystyrene xylene (DPX). Slides were scanned at 200 × magnification using a Nanozoomer Digital Pathology Scanner (Hamamatsu Photonics, Hertfordshire, UK). Staining was evaluated using a semi-quantitative H-score where the staining intensity (0 = absent, 1 = weak, 2 = strong) was multiplied by the percentage of tumour cells at each intensity. Over 30% of cores were independently scored, with an intraclass correlation coefficient of 0.700 indicating good inter-observer agreement.

### Statistics

IBM SPSS Statistics (V31.0) was used to perform statistical analysis. X-Tile software was used to order cases on the basis of survival (Camp et al. 2004). All differences of *P* ≤ 0.05 were stratified as statistically significant. To analyse the association between protein/mRNA expression and clinicopathological features, Pearson ꭕ^2^ test of association was used. Kaplan–Meier survival curves were plotted for analysis of survival outcomes with significance calculated using log-rank test.

To assess matched breast cancer tumour and adjacent normal tissue pairs, gene expression values were extracted from GDC for DHX37 and log2-transformed after adding a pseudocount of one. Differential expression between matched tumour and normal samples was assessed using paired statistical tests (paired t-test and Wilcoxon signed-rank test), with results considered statistically significant at *P* < 0.05. All analyses were performed in R (version RStudio 2025.05.1 + 513; R Foundation for Statistical Computing). TCGA RNA-seq data were obtained using the TCGAbiolinks package and processed using SummarizedExperiment. Gene expression values were log2-transformed prior to analysis. Statistical testing and visualisation were carried out in R using base statistical functions and the ggplot2 package.

## Results

### DHX37 protein expression in breast and ovarian cancer

**Fig. 1:**
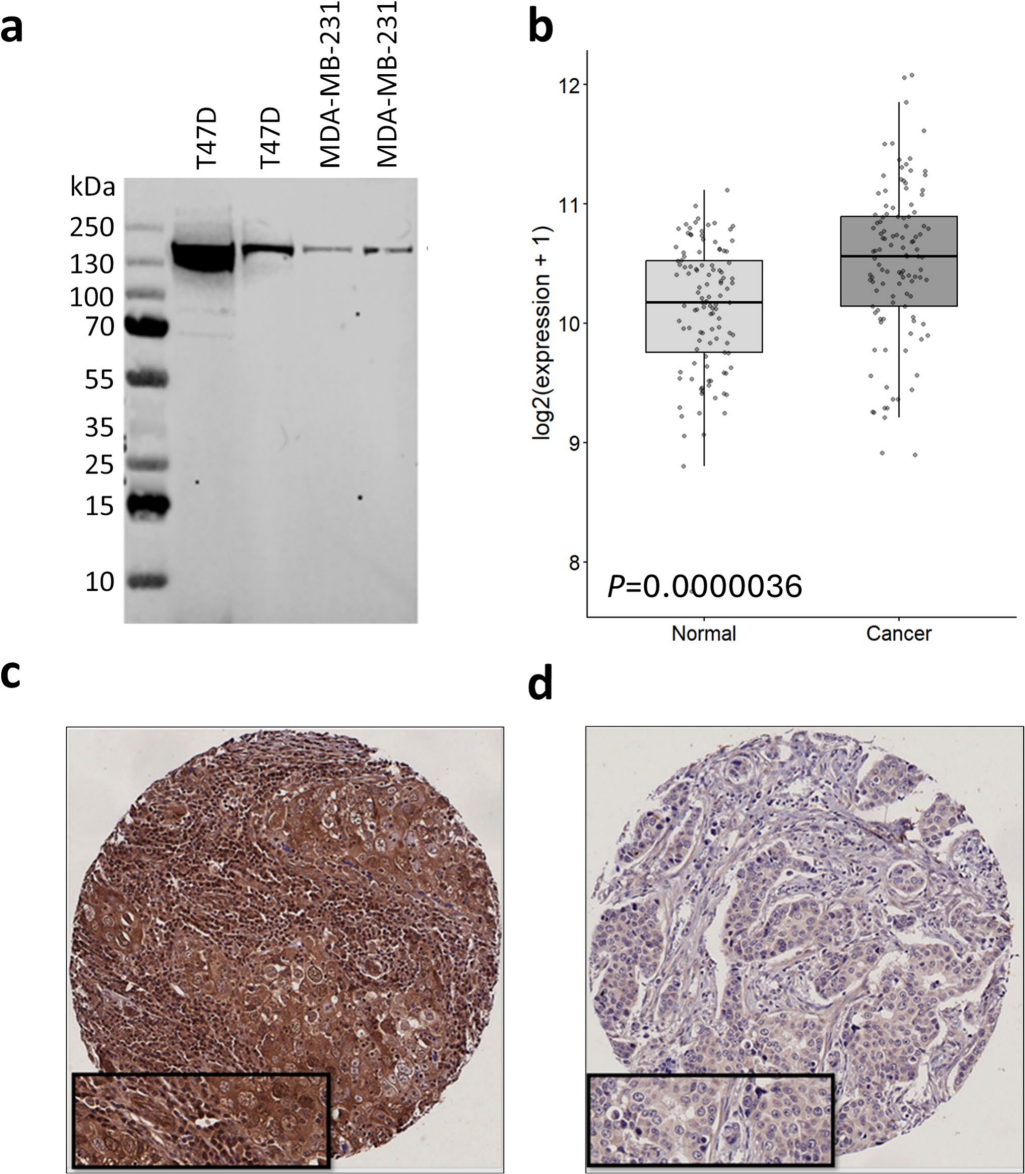
Western blotting of breast cancer cell lines utilising anti-DHX37 antibody against T47D and MDA-MB-231 cell lines and molecular weight ladder are shown in panel a. Box-and-whisker plot showing log2-transformed expression levels of DHX37 in matched primary tumour (TCGA-BRCA) and normal breast tissue samples is shown in panel b. Representative photomicrographs of DHX37 immunohistochemical staining in breast cancer, where (c) high staining and (c) low staining are shown. Photomicrographs were shown at 10 × magnification with 20 × magnification inset box

The specificity of the DHX37 antibody using Western blotting was confirmed in breast cancer cell lines (T47D and MDA-MB-231), prior to staining patient tissues (Fig. 1a); this antibody has been utilised in functional studies previously (Boneberg et al. 2019). To determine if there was a difference in DHX37 expression between normal and tumour tissue, gene expression was assessed in matched tumour-normal samples from the TCGA breast cancer cohort. DHX37 expression was significantly higher in primary tumour tissue compared with matched normal breast tissue (paired t-test, *P* < 0.001) (Fig. 1b).

The DHX37 antibody was used to stain 1512 early-stage breast tumours, with representative staining shown in Fig. 1c and d. The median cytoplasmic H-score in breast cancer was 172.5, ranging between 0 and 300. Using X-tile, a cut-off value of 180 was determined to dichotomise cases into high and low expression groups, with 50.1% (757/1512) of cases demonstrating low DHX37 expression.

In ovarian cancer (*n* = 420), the median cytoplasmic H-score was 200, ranging between 0 and 280. A cut point of 215, determined by X-tile, was used to classify cases into high and low expression categories. Low DHX37 expression was observed in 78.1% (328/420) of ovarian tumours.

### DHX37 protein expression and relationship with clinicopathological variables in breast cancer

The association between DHX37 protein expression and clinicopathological variables was examined. Low cytoplasmic DHX37 expression was significantly associated with larger tumour size (ꭕ^2^ = 17.228, d.f. = 1, *P* < 0.001), higher tumour grade (ꭕ^2^ = 26.27, d.f. = 2, *P* < 0.001), marked mitosis (ꭕ^2^ = 13.709, d.f. = 2, *P* < 0.001), positive lypmhovascular invasion (ꭕ^2^ = 11.255, d.f. = 1, P < 0.001), lower NPI prognostic group (ꭕ^2^ = 15.879, d.f. = 2,* P* < 0.001), negative PgR status (ꭕ^2^ = 13.237, d.f. = 1, *P* < 0.001), higher nuclear pleomorphism (ꭕ^2^ = 13.358, d.f. = 2, *P* < 0.001), less tubule formation (ꭕ^2^ = 30.770, d.f. = 2, *P* < 0.001) and positive lymph node involvement (ꭕ^2^ = 4.143, d.f. = 1, *P* < 0.001) (Table 1).

**Table 1:** Associations between DHX37 protein expression and clinicopathological criteria in breast cancer

	DHX37 protein expression		
	Low	High	*P* value
*Tumour Size*			
< 2 cm	432 (45.9%)	509 (54%)	** < 0.001**
≥ 2 cm	325 (56.9%)	246 (43.1%)	
*Grade*			
1	89 (36.0%)	158 (64.0%)	** < 0.001**
2	322 (50.4%)	317 (49.6%)	
3	346 (55.3%)	280 (44.7%)	
*Tubule Formation*			
1	31 (28.4%)	78 (71.6%)	** < 0.001**
2	218 (46.2%)	254 (53.8%)	
3	508 (54.6%)	423 (45.4%)	
*Pleomorphism*			
1	8 (30.8%)	18 (69.2%)	** < 0.001**
2	205 (44.5%)	256 (55.5%)	
3	544 (53.1%)	481 (46.9%)	
*Mitosis*			
1	350 (45.6%)	418 (54.4%)	** < 0.001**
2	175 (57.0%)	132 (43.1%)	
3	232 (53.1%)	205 (46.9%)	
*Lymph vascular Invasion*			
Absent	505 (47.3%)	563 (52.7%)	** < 0.001**
Present	252 (56.8%)	192 (43.2%)	
*Lymph node status*			
Negative	441 (47.9%)	479 (52.1%)	**0.042**
Positive	315 (53.3%)	276 (46.7%)	
*Nottingham prognostic index (NPI)*			
Good prognostic group	247 (44.0%)	314 (56%)	** < 0.001**
Medium prognostic group	371 (52.0%)	343 (48.0%)	
Poor prognostic group	138 (58.5%)	98 (41.5%)	
*Lymph node stage*			
Ln1	441 (47.9%)	479 (52.1%)	0.124
Ln2	233 (53.1%)	206 (46.9%)	
Ln3	82 (53.9%)	70 (46.1%)	
*Oestrogen receptor (ER) status*			
Negative	111 (50.5%)	109 (49.5%)	0.901
Positive	646 (50%)	646 (50%)	
*Progesterone receptor (PgR) status*			
Negative	292 (56.4%)	226 (43.6%)	** < 0.001**
Positive	458 (46.5%)	527 (53.5%)	
*HER2 status*			
Negative	675 (49.7%)	682 (50.3%)	0.456
Positive	82 (52.9%)	73 (47.1%)	
*Triple negative*			
Non-triple negative	662 (49.6%)	674 (50.4%)	0.529
Triple negative	84 (52.2%)	77 (47.8%)	
*Molecular class*			
Luminal A	281 (47.7%)	308 (52.3%)	0.077
HER2 enriched	16 (36.4%)	28 (63.6%)	
TNBC	84 (52.2%)	77 (47.8%)	
Luminal B	267 (53.2%)	235 (46.8%)	
*Age group*			
< 50 years	221 (48.5%)	235 (51.5%)	0.413
≥ 50 years	536 (50.8%)	520 (49.2%)	

Bold text indicates statistically significant associations

### DHX37 expression and relationship with breast cancer patient outcomes

**Fig. 2:**
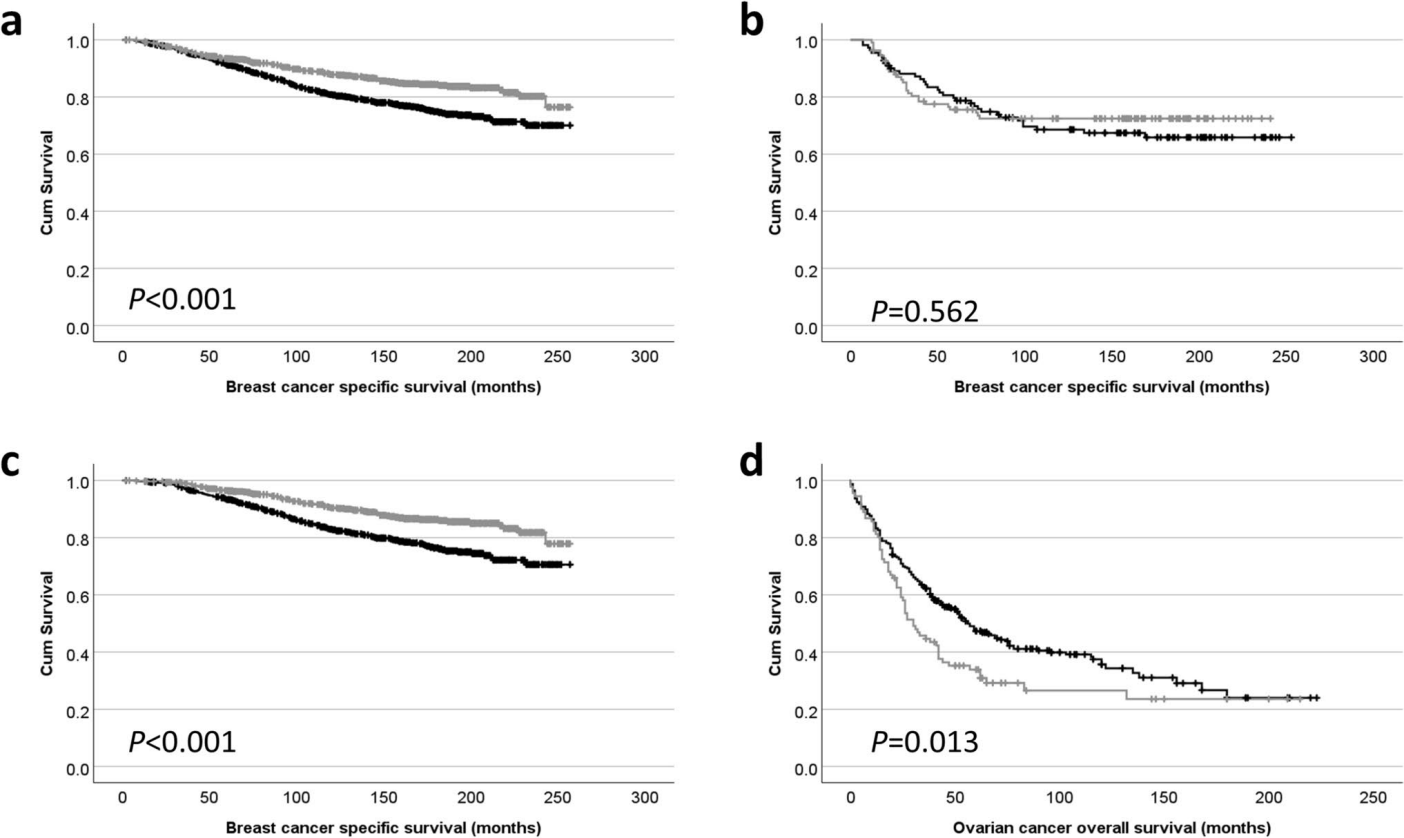
Kaplan–Meier analysis of breast cancer specific survival showing the impact of low (black line) and high (gray line) of DHX37 expression in **a** all patients, **b** ER positive patients and **c** ER negative patients. Kaplan–Meier analysis of ovarian overall survival showing the impact of low (black line) and high (gray line) DHX37 expression in **d** all patients

The association between cytoplasmic DHX37 protein expression and patient breast cancer-specific survival was investigated using Kaplan–Meier analysis. Low cytoplasmic DHX37 protein expression was significantly associated with adverse breast cancer-specific survival (*P* < 0.001) (Fig. 2a). The effect of DHX37 in patient subgroups was explored, with the most striking observation in ER positive patients (*P* < 0.001), in contrast to ER negative patients (*P* = 0.562) (Fig. 2b and c). Multivariate survival analysis was performed using Cox Regression and included tumour size, tumour grade, vascular invasion, ER status, PgR status and HER2 status, which were all individually associated with survival (*P* < 0.001). DHX37 expression was not independently associated with survival in this multivariate survival (hazard ratio (HR) = 0.617, 95% confidence interval (CI) = 0.617−1.004, *P* = 0.054).

### DHX37 protein expression and relationship with clinicopathological variables in ovarian cancer

The association between DHX37 protein expression in ovarian cancer and matched clinicopathological variables was assessed. Low DHX37 expression was associated with lower FIGO stage (ꭕ^2^ = 10.818, d.f. = 3, *P* = 0.013), no residual disease (ꭕ^2^ = 8.136, d.f. = 2, *P* = 0.017) and tumour histology (ꭕ^2^ = 14.874, d.f. = 5, *P* = 0.011) (Table 2).

**Table 2:** Associations between DHX37 protein expression and clinicopathological criteria in ovarian cancer

	DHX37 protein expression		
	Low	High	*P* value
*FIGO stage*			
1	124 (30.0%)	20 (4.8%)	**0.013**
2	37 (8.9%)	6 (1.4%)	
3	144 (34.8%)	51 (12.3%)	
4	22 (5.3%)	10 (2.4%)	
*Residual disease*			
No residual disease	191 (51.1%)	36 (9.6%)	**0.017**
Residual disease < 2CM	35 (9.4%)	12 (3.2%)	
Residual disease > 2CM	71 (19.0%)	29 (7.8%)	
*Tumour grade*			
1	27 (6.4%)	6 (1.4%)	0.170
2	56 (13.4%)	9 (2.1%)	
3	244 (58.2%)	77 (18.4%)	
*Histology*			
High grade serous	192 (45.7%)	69 (16.4%)	**0.011**
Mucinous	41 (9.8%)	4 (1.0%)	
Endometrioid	39 (9.3%)	7 (1.7%)	
Clear-cell	36 (8.6%)	3 (0.7%)	
Low grade serous	12 (2.9%)	5 (1.2%)	
Borderline serous	8 (1.9%)	4 (1.0%)	

Bold text indicates statistically significant associations

### DHX37 protein expression and relationship with ovarian cancer patient outcomes

The association between cytoplasmic DHX37 protein expression and overall survival of patients with ovarian cancer was investigated using Kaplan–Meier analysis. Low DHX37 protein expression was associated with improved ovarian cancer survival (*P* = 0.013) (Fig. 2d). Multivariate survival analysis was performed using Cox Regression including grade, FIGO stage, histological subtypes and the presence of residual disease. DHX37 expression was not independently associated with survival in this multivariate survival analysis (HR = 1.105, 95%, CI = 0.796–1.534, *P* = 0.550).

### DHX37 mRNA expression and its relationship with clinicopathological variables and patient survival in breast and ovarian cancer in the TCGA datasets

The expression of *DHX37* mRNA within the TCGA pan cancer atlas breast (*n* = 1082) and ovarian (*n* = 300) cancer cohorts (BRCA and OVCA) and their association with clinicopathological criteria were assessed.

**Fig. 3:**
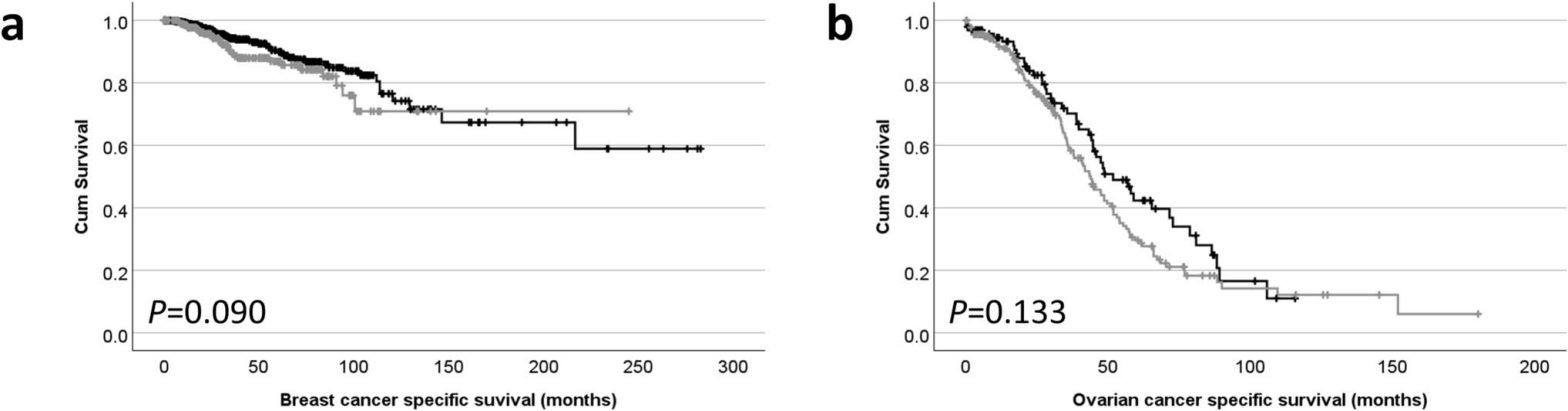
Kaplan–Meier analysis of cancer specific survival showing the impact of low (black line) and high (gray line) *DHX37* mRNA expression in **a** breast cancer and **b** ovarian cancer

In the TCGA breast cancer cohort, *DHX37* had a median *Z*-score of –0.1481 (ranging from –0.1481 to 23.46). X-tile was used to dichotomise data into low and high expression with the cut point being 0.17, with 689 (63.7%) having low expression. Low *DHX37* expression was associated with Luminal B molecular subtype tumours (ꭕ^2^ = 33.775, d.f. = 4, *P* < 0.001), no other associations were observed (Table 3). *DHX37* mRNA expression in the breast cancer cohort was not associated with patient survival (*P* = 0.090) (Fig. 3a).

**Table 3:** Associations between DHX37 mRNA expression and clinicopathological criteria in breast cancer

	*DHX37* mRNA expression		
	Low	High	*P* value
*AJCC tumour stage*			
1	176 (16.3%)	100 (9.3%)	0.98
2	401 (37.2%)	226 (20.9%)	
3	85 (7.9%)	52 (4.8%)	
4	25 (2.3%)	14 (1.3%)	
*AJCC node stage*			
0	320 (30.1%)	192 (18.1%)	0.235
1	235 (22.1%)	120 (11.3%)	
2	80 (7.5%)	39 (3.7%)	
3	42 (4.0%)	34 (3.2%)	
*Molecular subtype*			
Basal	97 (9.9%)	74 (7.5%)	** < 0.001**
HER2	41 (4.2%)	37 (3.8%)	
LumA	355 (36.2%)	144 (14.7%)	
LumB	115 (11.7%)	82 (8.4%)	
Normal	13 (1.3%)	23 (2.3%)	

Bold text indicates statistically significant associations

In the TCGA ovarian cancer cohort, *DHX37* had a median *Z*-score of –0.0964 (ranging from –2.9286 to 13.4367). X-tile was used to dichotomise data into low and high expression with the cut point being –0.72, with 108 (36.0%) having low expression. *DHX37* expression was not associated with tumour stage (ꭕ^2^ = 4.620, d.f. = 7, *P* = 0.706) or tumour grade (ꭕ^2^ = 4.840, d.f. = 2, *P* = 0.089). *DHX37* mRNA expression in the ovarian cancer cohort was not associated with patient survival (*P* = 0.133) (Fig. 3b).

## Discussion

DEAD/DEAH box helicases are recognised as key regulators of tumour biology owing to their role in RNA metabolism, ribosome biogenesis and immune modulation. Several helicases, including DDX5, DDX21 and DHX9, have been shown to contribute to cancer progression by promoting cell proliferation, regulating oncogenic transcription and suppressing antitumour immune responses (Fuller-Pace 2013; Liu et al. 2014; Murayama et al. 2024).

In the current study, in breast cancer, low cytoplasmic DHX37 protein expression was significantly associated with adverse breast cancer-specific survival (*P* < 0.001), particularly among ER-positive patients, whereas no association was observed in ER-negative cases. By contrast, low DHX37 protein expression in ovarian cancer was significantly associated with improved overall survival (*P* = 0.013). Analysis of *DHX37* mRNA expression in TCGA datasets for both breast and ovarian cancer revealed no significant association with patient survival (*P* = 0.090 and *P* = 0.133, respectively). These contrasting associations in breast and ovarian cancer suggest that DHX37 plays context-dependent roles in tumour biology, acting as a tumour suppressor in breast cancer, whilst potentially exerting oncogenic effects in ovarian cancer. These context-dependent effects may be explained by the involvement of DHX37 in RNA metabolism and innate immune signalling, with different interactions with ER-pathways and the tumour immune microenvironment, that may drive divergent roles in breast and ovarian cancer. This study has focused on evaluating DHX37 expression in tumour tissue alone, without assessing differences relative to matched normal tissues.

These findings align with the results of the present study, where low DHX37 protein expression in breast cancer was associated with adverse clinicopathological features and poorer survival. Conversely, in ovarian cancer, low DHX37 expression was associated with better clinicopathological variables and improved survival. This highlights a context-dependant role similar to other DEAH-box helicases, which can act as oncogenic drivers in some tissues but may have tumour suppressive functions in others (Fuller-Pace 2013). Collectively, these findings support the notion that DEAH-box helicases, including DHX37, integrate transcription regulation and repress innate immunity to influence cancer features and outcomes.

In breast cancer, low DHX37 protein expression was associated with larger tumour size, higher tumour grade, lymphovascular invasion and positive nodal involvement. These findings suggest that loss of DHX37 may contribute to tumour proliferation and invasiveness, particularly within ER positive subtypes. By contrast, a pan-cancer bioinformatic analysis utilising TCGA and GTEx datasets reported that high DHX37 mRNA expression was linked to worse overall survival across multiple tumour types, including breast cancer, suggesting that it may act as an oncogenic driver (Huang et al. 2021). This discrepancy may reflect differences in molecular level assessed as mRNA expression does not always correlate with protein abundance or activity. Furthermore, post-transcriptional regulation and tumour micro-environmental factors may influence the functional role of DHX37.

These data indicate that DHX37 could serve as a prognostic biomarker, particularly in ER positive breast cancer, where low expression identifies patients with poorer outcomes. Given its multifunctional roles in RNA metabolism and immune modulation, DHX37 may also represent a therapeutic target in certain tumour types, and helicase inhibition could be explored as a strategy to restore antitumour immunity or disrupt tumoural RNA processing. Future work should include in vitro and in vivo studies to delineate these mechanisms and validate the functional role of DHX37 in cancer progression.

In summary, this study identifies DHX37 as a context-dependent regulator of tumour biology in breast and ovarian cancer. Further integrated molecular, immunological and functional analyses will be essential to clarify how DHX37 contributes to tumourigenesis and to assess its utility as a prognostic and therapeutic target across multiple cancer types.

## Data Availability

The mRNA expression data presented in this study are available for download (http://www.cbioportal.org), with immunohistochemistry data available on request.

## Abbreviations

CI: Confidence interval
DSD: Disorders of sexual development
DHX37: DEAH-box RNA helicase 37
ER: Oestrogen receptor
HR: Hazard ratio
PgR: Progesterone receptor
